# The Resistance of Germinating Pea (*Pisum sativum* L.) Seeds to Silver Nanoparticles

**DOI:** 10.3390/plants14111594

**Published:** 2025-05-23

**Authors:** Karolina Stałanowska, Katarzyna Głowacka, Bogusław Buszewski, Lesław Bernard Lahuta

**Affiliations:** 1 Department of Plant Physiology, Genetics and Biotechnology, University of Warmia and Mazury in Olsztyn, Oczapowskiego 1A, 10-719 Olsztyn, Poland; katarzyna.glowacka@uwm.edu.pl; 2 Department of Environmental Chemistry and Bioanalytics, Faculty of Chemistry, Nicolaus Copernicus University, Gagarina 7, 87-100 Toruń, Poland; bbusz@chem.umk.pl; 3 Professor Jan Czochralski Kuyavian-Pomeranian Scientific Technological Centre, Krasińskiego 4, 87-100 Toruń, Poland

**Keywords:** pea, seedling, silver nanoparticles, ROS, polar metabolites

## Abstract

The results of our recent research revealed that biologically synthesized silver nanoparticles (bio-AgNPs) applied to several-day-old pea (*Pisum sativum* L.) plants or used for seed nanopriming protected pea plants against selected fungal pathogens. However, the susceptibility of pea to bio-AgNPs during seed germination remains mostly unknown. Therefore, in this study, we investigated the cells’ viability, ROS generation, total antioxidant capacity, the activity of selected antioxidant enzymes, and changes in the polar metabolite profiles of 4-day-old pea seedlings developed in water (control) and water suspensions of bio-AgNPs (at 50 and 200 mg/L). The bio-AgNPs did not negatively affect pea seeds’ germination, early seedlings’ growth, and root tips cells’ viability (at both tested concentrations). In the root, the bio-AgNPs at a lower concentration (50 mg/L) stimulated ROS generation. Nanoparticles enhanced peroxidase activity in root and the total antioxidant capacity in epicotyl. Increased levels of malate, phosphoric acid, proline, GABA, and alanine were observed in root and epicotyl of pea seedlings developed at 50 mg/L of bio-AgNPs. A higher concentration affected the tricarboxylic acid cycle and nitrogen metabolism. Bio-AgNPs alerted oxidative homeostasis and primary metabolism of pea seedlings but did not exceed a certain threshold limit and thus did not injure pea at an early stage of seedling development.

## 1. Introduction

Nanoparticles (NPs), especially the metallic/metalloid nanoparticles, are widely used in many industrial sectors: electronics, textiles, and environmental remediation, as well as in cosmetics, medicine, and agriculture [1,2,3]. The scope of NP applications is significantly influenced by the method of their synthesis. Nanoparticles can be manufactured chemically, physically, and biologically. Biological synthesis methods, so-called “green methods”, are more eco-friendly, economical, and sustainable than other methods, which utilize various chemicals or have significant energy expenditure [3,4,5,6]. In the production of NPs, hazardous reagents are replaced with microorganisms, post-culture or post-fermentation medium, or plant extracts used as both reducing and stabilizing agents. This approach impacts nanoparticles’ characteristics, enhancing their biocompatibility [6,7,8], which as a result can make them less toxic to living organisms [3,6,7,9,10].

Currently, among the most utilized nanoparticles are silver nanoparticles (AgNPs). Due to their unique optical, conductive, and antimicrobial properties, AgNPs are used in electronics, textiles, medical equipment, or food packaging [2,8,11]. Moreover, they have great potential for agricultural applications such as nano-pesticides and nano-fertilizers [11]. However, the effect of AgNPs, as with other metallic nanoparticles, on plants can be both phytotoxic and growth-stimulatory, which depends on the plant’s species, the type and physicochemical properties of NPs, as well as their concentration [1,3].

The dose-dependent effects of silver nanoparticles have been well-documented, e.g., for cowpea beans (*Vigna unguiculata* L.), where AgNPs at concentrations of 10 to 60 mg/L stimulated germination and root length, while at higher concentrations (80 and 100 mg/L), they had an inhibiting effect [12]. Similarly, Koley et al. [13] demonstrated that AgNPs at lower concentrations (10 and 50 mg/L) stimulated the germination and seedling length of pea (*Pisum sativum* L.), chickpea (*Cicer arietinum* L.), and mung bean (*Vigna radiata* (L.) R. Wilczek), but at a higher dose (100 mg/L), had the opposite effect. Our previous research showed that AgNPs at a concentration of 20 mg/L and Ag^+^ ions (in form of silver nitrate) at concentrations of 20 and 50 mg/L stimulated pea seedlings’ growth, but much higher concentrations of AgNO_3_ (500 and 1000 mg/L) negatively affected pea seeds’ germination and seedlings’ growth [14].

The toxicity of AgNPs is usually connected to morpho-anatomical changes, DNA damage, and generation of reactive oxygen species (ROS). Subsequently, ROS causes lipid peroxidation and an uncontrolled increase in cell membrane permeability, which lead to the disruption of cell metabolism and cell death [2,15]. Our team presented that bio-AgNPs, synthesized with a *Lactobacillus paracasei* post-culture medium [16], are toxic to wheat (*Triticum aestivum* L.) seedlings when applied at concentrations of 20 mg/L and higher, which is connected to the generation of ROS in the root growth tip/root cap [17]. A similar reaction in the root tips of wheat was noted for chemically synthesized AgNPs, especially in the case of nanoparticles with sizes of 10 and 20 nm, which was accompanied by a decrease in the primary root length [18]. Thus, it can be expected that plant exposure to AgNPs also stimulates antioxidant systems. Indeed, an increase in antioxidative enzyme activities and total antioxidant capacity in a dose-dependent manner was observed in sage (*Salvia officinalis* L.) treated with AgNPs [19]. Panda et al. [20] showed that the toxicity of AgNPs to onion (*Allium cepa* L.) is mediated by ROS generation. ROS in excess can be harmful to plant cells, but in moderate amounts can act as signaling molecules, crucial for plant growth, development, and defense [21]. This suggests that AgNPs can boost many processes in plants by accurately triggering ROS production when used at appropriate concentrations for particular plant species. However, crossing that line leads directly to disruption in plants’ organisms.

The results of our recent research revealed that bio-AgNPs can be used as an effective agent for pea plant protection against some fungal pathogens, i.e., *Didymella pinodes* and *Fusarium avenaceum*, causing Ascochyta blight and Fusarium root rot diseases, respectively [22,23]. The application of bio-AgNPs to pea seedlings or for seed priming successfully reduced the infection of pathogens and was safe for several-day-old pea plants. However, the mechanism of pea resistance to bio-AgNPs remains unknown. Therefore, in this study, we focused on the effect of bio-AgNPs on cell viability, ROS generation, total antioxidant capacity, the activity of selected antioxidant enzymes, as well as changes in the polar metabolite profiles in root, epicotyl, and cotyledons during pea seedling early development.

## 2. Results

### 2.1. Effects of Bio-AgNPs on Seedlings’ Growth

Pea seeds’ germinability, seedlings’ length, and the FW and DW of roots and epicotyls after 4 days of seed germination in the presence of biologically synthesized silver nanoparticles (bio-AgNPs) at concentrations of 50 and 200 mg/L were not affected in comparison to the control (seed germinated in the water). However, there are some slight trends/tendencies of stimulation of the root’s length and its fresh weight and dry weight by nanoparticles. No negative effects on the seedlings’ FW and length of two other pea cultivars (‘Tarchalska’ and ‘Sześciotygodniowy Tor’) were observed (Table S1). Thus, the germinating pea seeds and early developing seedlings seem to be resistant to bio-AgNPs at the tested concentrations (Figure S1A, Table 1).

The effects of bio-AgNPs on the germination and seedling growth of wheat, radish (*Raphanus sativus* L.), and cress (*Lepidium sativum* L.) were tested, additionally, for a comparison of the resistance of the mentioned species to bio-AgNPs with that of pea. The results revealed the phytotoxicity of bio-AgNPs to wheat (Figure S1B, Table S2) and cress (Figure S1D, Table S2). In both species, the length of 4-day-old seedlings was significantly reduced compared to the control. Moreover, the additional test revealed that the bio-AgNPs in the range of 25–100 mg/L stimulated radish growth (both root and hypocotyl; Figure S1C, Table S2), whereas increasing the concentration up to 200 mg/L leads to ca. 50% inhibition of seedling elongation (Table S2). The maximum seedling length and fresh weight were observed at a concentration of 75 mg/L. Higher tested concentrations significantly reduced the root length (150 and 200 mg/L) and hypocotyl length (200 mg/L; Table S2). Seedlings of cress were more susceptible to bio-AgNPs, and a reduction in seedling length was observed just at a concentration of 25 mg/L. The increasing concentration of bio-AgNPs significantly reduced seedling growth, by ca. 50% at concentrations of 150–200 mg/L (Table S2). Thus, the resistance/susceptibility of germinating seeds to bio-AgNPs seems to be plant species-dependent.

### 2.2. Effect of Bio-AgNPs on Seedlings’ Viability and Antioxidant System

**Figure 1:**
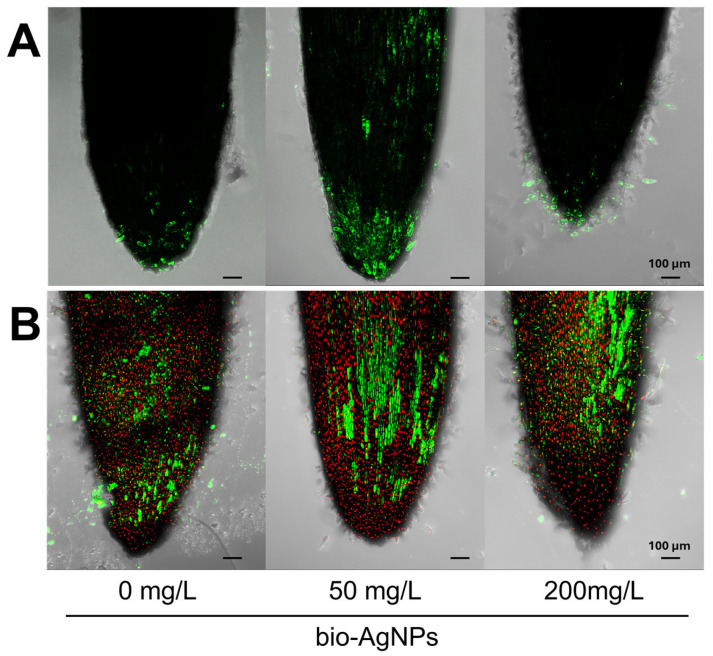
Presence of (**A**) reactive oxygen species (ROS) and (**B**) viable (green) and dead (red) cells in root tips of 4-day-old pea (*Pisum sativum* L.) seedlings developed in suspension of bio-AgNPs at concentrations of 0, 50, and 200 mg/L. Scale bars equal 100 µm.

Bio-AgNPs at a concentration of 50 mg/L stimulated the generation of ROS in cells of the root cap of pea seedlings (and to a lesser extent in cells of the root apex, just above the apical meristem), but there was no such evidence at a higher tested concentration, where the ROS level was similar to the control, concentrated at the root cap (Figure 1A). Moreover, nanoparticles at both tested concentrations did not negatively affect the viability of root tip cells (Figure 1B), which, along with the unaffected pea seedling size (Table 1), confirmed the non-harmful effect of bio-AgNPs.

**Figure 2:**
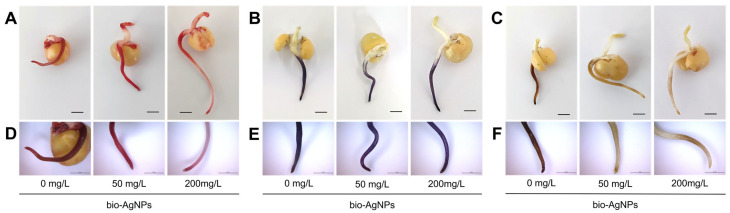
Histochemical staining of 4-day-old pea (*Pisum sativum* L.) seedlings developed in suspension of bio-AgNPs, with (**A**) TTC to determine the cells’ viability, (**B**) with NBT to determine the localization of O_2_^−^, and (**C**) with DAB to determine the localization of H_2_O_2_. The bottom panels show enlarged fragments of the roots after staining with (**D**) TTC, (**E**) NBT, and (**F**) DAB. Horizontal scale bars equal 5 mm.

TTC staining showed a high respiration intensity in the root tips at all tested bio-AgNP concentrations (Figure 2A,D), indicating high cell viability, which was consistent with the results for the presence of live/dead cells (Figure 1B). However, bio-AgNPs at the concentration of 200 mg/L caused unequally intensive respiration along the entire root length, which was not observed at the concentration of 50 mg/L or in the control. The presence of reactive oxygen species in roots was confirmed by NBT and DAB staining. The NBT staining intensities showed that O_2_^−^ radicals were present at similar levels in the roots, regardless of the nanoparticle concentration (Figure 2B,E). The H_2_O_2_ level, localized with DAB, was lower in roots of pea seedlings developed in nanoparticle suspensions than in the control (Figure 2C,F).

Moreover, the DAB staining intensity decreased with an increasing nanoparticle concentration. This may have affected the fluorescent visualization of ROS. The standard procedure for determining the presence of ROS uses 2,7-dichlorodihydrofluorescein diacetate (H_2_DCF-DA), which is oxidized by reactive oxygen species to fluorescent 2,7-dichlorofluorescein (DCFH_2_). This probe is typically used as a qualitative marker for the total ROS as it is non-specific. H_2_DCF-DA shows reactivity with H_2_O_2_, hydroxyl radical (˙OH), or singlet oxygen (^1^O_2_) but no or little reactivity with O_2_^−^ [24]. Thus, a lesser extent of H_2_O_2_, with an unchanged presence of O_2_^−^, could cause a lower fluorescent signal in roots. This might also suggest a lower level of the other ROS, like ˙OH or ^1^O_2_.

We also investigated the activity of selected antioxidative enzymes in pea seedlings. Increased activity of ascorbate peroxidase (APX) was observed in the root and decreased activity was noted in the epicotyl of seedlings developed in bio-AgNPs at both concentrations (Table 2). Guaiacol peroxidase (GPOX) activity decreased in all tissues of pea seedlings grown in the presence of bio-AgNPs (roots only at 200 mg/L) (Table 2).

Moreover, in the epicotyl as well as in roots of seedlings developed in bio-AgNPs, changes in activities of catalase (CAT) and SOD were not significantly affected. Thus, the more intense decomposition of H_2_O_2_ in the root of seedlings developed in bio-AgNPs, as shown by DAB staining (Figure 2C,F), was caused by ascorbate peroxidase. The lack of changes in SOD activity was consistent with NBT staining (Figure 2B,E), indicating that bio-AgNPs did not contribute to the excessive generation of superoxide radicals. In cotyledons, an evident decrease in CAT activity and increase in SOD (at 50 mg/L of bio-AgNPs) were noted (Table 1). The total antioxidant capacity (TAC) was elevated in the epicotyl in the presence of bio-AgNPs, which was not observed in the root and cotyledons (Table 1).

### 2.3. Polar Metabolites Profiles

#### 2.3.1. Metabolic Profile of Control Seedlings

In tissues of 4-day-old pea seedlings, 36 metabolites were identified, including 9 soluble carbohydrates (fructose, galactose, glucose, galactinol, *myo*-inositol, raffinose, stachyose, sucrose, gluconic acid), 17 amino acids (alanine, asparagine, aspartic acid, β-alanine, γ-aminobutyric acid (GABA), glutamic acid, homoserine, hydroxyproline, isoleucine, leucine, lysine, phenylalanine, proline, serine, threonine, tyrosine, valine), 8 organic acids (citric acid, fumaric acid, lactic acid, malic acid, malonic acid, oxalic acid, propionic acid, succinic acid), and phosphoric acid urea (Tables S3 and S4).

The contents of total identified polar metabolites (TIPMs) in the epicotyl (142.84 mg/g DW) and root (134.33 mg/g DW) of the control seedlings were about 2-fold higher than in cotyledons (77.47 mg/g DW). The most abundant fractions in developing tissues were soluble carbohydrates (SCs) and amino acids (AAs), ranging from 40 to 50% and from 35 to 40% of TIPMs, respectively (Tables S3 and S4). The dominant metabolites were sucrose, homoserine, phosphoric acid, and malate. In storage tissue, soluble carbohydrates dominated (sharing 80% of TIMPs), with sucrose as the metabolite with the highest concentration (Table S5). Moreover, galactinol, raffinose, and stachyose were detected only in cotyledons.

#### 2.3.2. Changes in Metabolic Profiles After Exposure to Bio-AgNPs

**Figure 3:**
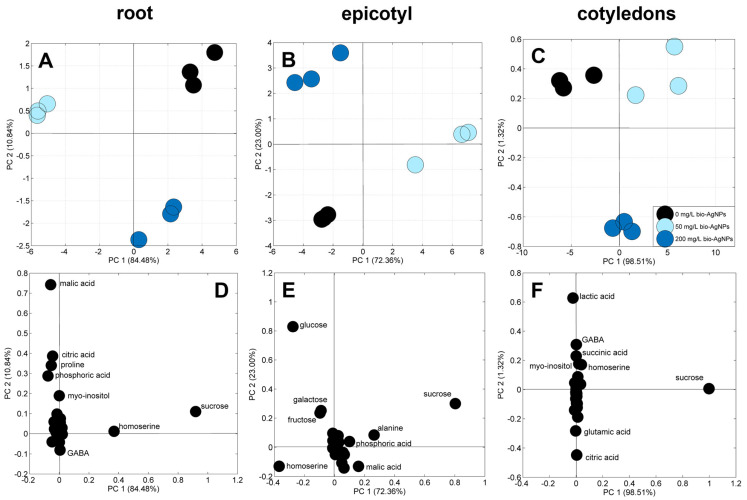
PCA score (**A**–**C**) and loading plots (**D**–**F**) of the metabolic profiles of the root (**A**,**D**), epicotyl (**B**,**E**), and cotyledons (**C**,**F**) of 4-day-old pea (*Pisum sativum* L.) seedlings developed in suspensions of bio-AgNPs at concentrations of 0, 50, and 200 mg/L. Legend for plots (**A**–**C**): black, light blue, and dark blue circles refer to data of tissues of seedlings developed in 0, 50, and 200 mg/L of bio-AgNPs, respectively.

The principal component analysis (PCA) of the polar metabolites of root, epicotyl, and cotyledons revealed clear data separation (Figure 3A–C).

The samples of seedlings grown in bio-AgNPs at concentrations of 0, 50, and 200 mg/L were separated from each other according to both PC1 and PC2 in the root (PC1—84.48%; PC2—10.84%), epicotyl (PC1—72.36%; PC2—23.00%), and cotyledons (PC1—98.51%; PC2—1.32%). Moreover, in root and cotyledons, the control and concentration of 50 mg/L of bio-AgNPs were divided from the concentration of 200 mg/L according to PC2, sharing 10.84% and 1.32% of variability, respectively.

According to the loading plots, the distribution of the root and epicotyl samples was mainly related to differences in the concentrations of sucrose, homoserine, malic acid, and phosphoric acid (Figure 3D,E). Additional differentiating metabolites in roots were citric acid, *myo*-inositol, proline, and GABA, whereas in epicotyls, they were alanine and monosaccharides like glucose, galactose, and fructose. Sucrose and homoserine were also the main differentiating metabolites in cotyledons samples, as well as citrate, lactate, succinate, *myo*-inositol, glutamic acid, and GABA (Figure 3F).

Seed germination and subsequent seedling development in bio-AgNPs caused changes in concentrations of identified polar metabolites. In the root of seedlings developed in bio-AgNPs, the TIPM level decreased (from 134 to 124 mg/g DW), whereas in epicotyl and cotyledons, it slightly (by about 5–10%) increased (from 143 to ca. 150 and from 77 to 87 mg/g DW, respectively; Tables S3–S5).

### Soluble Carbohydrates

**Figure 4:**
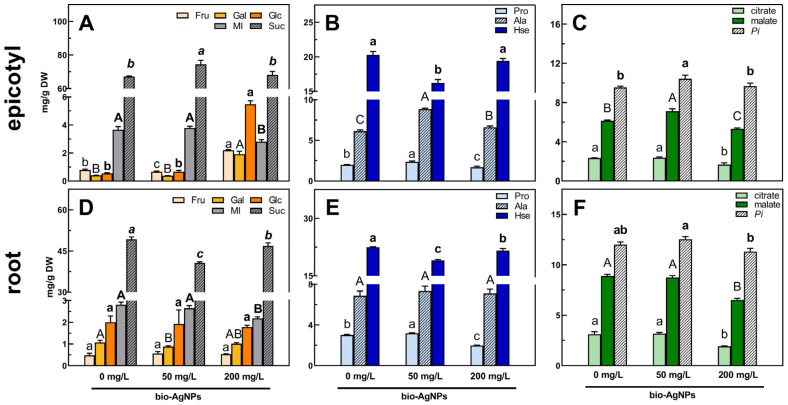
The concentrations of selected metabolites in the epicotyl (**A**–**C**) and root (**D**–**F**) of 4-day-old pea (*Pisum sativum* L.) seedlings developed in suspension of bio-AgNPs at concentrations of 0, 50, and 200 mg/L. Values (in mg/g DW) are the means of 3 replicates + SD. The same letters (a–c; A–C; **a**–**c**; **A**–**B**; ***a****–**c***; separately for each metabolite) above the bars indicate no statistically significant (*p* ≤ 0.05) differences based on ANOVA and Tukey’s post hoc test. Abbreviations: Fru—fructose; Gal—galactose; Glc—glucose; MI—*myo*-inositol; Suc—sucrose; Pro—proline; Ala—alanine; Hse—homoserine; *Pi*—phosphoric acid.

Changes in the content of TIMPs were mostly a result of changes in the concentration of soluble carbohydrates as fraction of metabolites with the highest concentration. The total soluble carbohydrate (TSC) content decreased in the root (Table S3) and increased in epicotyl (Table S4) and cotyledons (Table S5) of seedlings developed in bio-AgNPs. This is consistent with the changes in the content of sucrose (the quantitatively predominant sugar), which decreased in roots (Figure 4D; Table S3), but increased in epicotyls (Figure 4A; Table S4) and cotyledons (Figure S3A; Table S5).

In root, the decrease in sucrose level was not accompanied by a change in fructose or glucose, but only a decrease in the content of *myo*-inositol (seedlings developed at 200 mg/L of bio-AgNPs) and galactose (developed at 50 mg/L of bio-AgNPs; Figure 4D, Table S3). A similar decrease in *myo*-inositol content was also noted in the epicotyl at the highest tested nanoparticle concentration (Figure 4A, Table S4). In epicotyl, the highest sucrose content (74.36 mg/g DW) and lowest fructose content (0.66 mg/g DW) were observed for seedlings developed at 50 mg/L of bio-AgNPs compared to control seedlings (67.17 mg/g DW of sucrose; 0.76 mg/g DW of fructose). In the case of seedlings developed at 200 mg/L, almost 3-fold and over 5-fold and 10-fold increases in fructose (to 2.20 mg/g DW), galactose (to 1.91 mg/g DW), and glucose (to 5.48 mg/g DW) contents, respectively, were noted but the sucrose content remained unchanged (Figure 4A, Table S4). In cotyledons of seedlings grown at 50 mg/L of bio-AgNPs, the increased content of *myo*-inositol and absence of stachyose were observed, whereas at 200 mg/L, increased contents of raffinose and galactinol were noted (Table S5).

### Amino Acids

The overall level of total amino acids (TAAs, calculated as a sum of the contents of all amino acids identified) did not change in the root and epicotyl (Tables S3 and S4). However, in those tissues, similar changes in the contents of homoserine (Hse) and proline (Pro) were observed. The level of Hse decreased in seedlings developed in bio-AgNPs at 50 mg/L (16.22–19.04 mg/g DW), while at 200 mg/L, it was as high as in the control seedlings (in epicotyl—19.41 mg/g DW; Figure 4B; Table S4) or slightly lowered, but still higher than at 50 mg/L (in root—21.61 mg/g DW; Figure 4E; Table S3).

In pea root and epicotyl, the content of proline increased significantly at a concentration of 50 mg/L of bio-AgNPs (2.37–3.19 mg/g DW), while it decreased at 200 mg/L (1.70–1.97 mg/g DW; Figure 4B,E; Tables S3 and S4). Moreover, at the highest concentration of nanoparticles, the GABA content increased in developing tissues (in epicotyl not significantly), which was accompanied by a decrease in glutamate (Glu) content (Tables S3 and S4).

Alanine (Ala) was also considered a differentiated metabolite in the epicotyl samples according to the PCA loading plots (Figure 3E). The content of Ala increased in the epicotyl of seedlings developed in nanoparticle suspensions, and its highest concentration was observed for 50 mg/L of bio-AgNPs (8.86 mg/g DW; Figure 4B; Table S4).

In contrast to epicotyls and roots, the TAA content in cotyledons of seedlings developed in bio-AgNPs increased (from 8.80 to 9.31–9.53 mg/g DW) due to the elevated levels of certain amino acids (i.e., glutamate, asparagine, leucine, isoleucine, serine; Table S5). Opposite to roots, the contents of GABA and Ala lowered (Figure S2C; Table S5).

### Organic Acids and Remaining Compounds

The total organic acid (TOA) content did not change in the root (Table S2) and increased in the epicotyl of seedlings developed at 50 mg/L of bio-AgNPs, mainly due to the elevated malate content (Table S4; Figure 4C). In seedings developed at 200 mg/L of bio-AgNPs, the TOAs significantly decreased, due to the reduction in citrate and malate levels (Figure 4C,F; Tables S3 and S4). In the cotyledons, large decreases in lactate (by 70%) and succinate (by 40%) were noted, with an increase in the contents of citric and malic acid (Figure S2C; Table S5).

The highest content of phosphoric acid (*Pi*) was noted in all tissues of seedings developed in the bio-AgNP suspension at a concentration of 50 mg/L (Figure 4C,F and Figure S2C; Tables S3 and S4).

## 3. Discussion

### 3.1. Species-Specific Effect of Bio-AgNPs on Seedling Growth

Pea seeds’ germinability and seedlings’ development in the bio-AgNP suspensions were not affected (Figure S1A, and ). Thus, the germinating pea seeds and early developing seedlings seem to be resistant to bio-AgNPs at the tested concentrations, which is in contrast to the results of our previous studies on wheat germinated in the presence of the same bio-AgNPs, applied at even lower concentrations of 20–40 mg/L [17].

The resistance of germinating seeds/seedlings to AgNPs (applied at 50 mg/L) was found among various species: tomato (*Solanum lycopersicum* L.), kale (*Brassica oleracea* L. var. *sabellica* L.) [25], chickpea (*Cicer arietinum* L.), and mung bean (*Vigna radiata* L.) [13]. In the case of pea, AgNPs synthesized with glucose and gelatin (spherical in shape), applied at low concentrations (20 and 40 mg/L), did not negatively affect seedling growth [26], similar to AgNPs synthesized with an aqueous extract of *Parthenium hysterophorus* L. [13]. Moreover, the early growth of pea seedlings can be slightly stimulated by citrate-stabilized spherical AgNPs (at 20 mg/L) [14] as well as by triangular saponin-capped AgNPs but applied at much lower concentrations (2.5–8 µg/L) [27]. However, AgNPs at higher concentrations (80 and 160 mg/L) can cause serious damage, such as mitosis disturbance, chromosomal aberrations [26], cell plasmolysis, vacuolization, and structural malformations in mitochondria and chloroplasts [28].

We additionally tested the potentially phytotoxic effect of bio-AgNPs on the germination and seedling growth of other species: wheat, radish, and cress. Radish and cress, members of the Brassicaceae family, annual and fast-growing herbs widely consumed all around the world [29,30], were proposed as model plants for the study of environmental stresses and pollution [31,32,33]. Our results, revealing the stimulatory effects of bio-AgNPs up to 100 mg/L on radish seedling growth (Table S2), are consistent with previous findings by Tymoszuk [25], who showed that chemically synthesized AgNPs (2 nm in size; at 50 and 100 mg/L) stimulated the root length of radish seedlings during 3 weeks of growth in a medium with nanoparticles. However, toxic effects of chemically synthesized AgNPs on germinating radish seeds and developing seedlings were found, and it was demonstrated that not only the size but also different surface coatings of AgNPs had a great impact on their biological activity [31,34]. In our study, increasing the concentration of bio-AgNPs up to 150 mg/L also significantly (*p* < 0.05) inhibited seedling growth (Table S2). A similar susceptibility of radish sprouts to AgNPs at concentrations above 125 mg/L was documented by Zuverza-Mena et al. [31]. However, our results revealed a hormetic (a positive or negative dose response above a specific concentration) effect of bio-AgNPs on radish seedling’s growth. The hormetic responses to AgNPs were also found in some bacteria, algae, and plants [35,36].

Bazoobandi et al. [37] showed that AgNO_3_ and nanoparticles with positive surface charge (coated with polyethyleneimine) had more negative impact on shoot and fine roots dry weight of radish than negatively charged AgNPs (coated with citrate and PVP) at the same tested concentrations (25 and 125 mg/kg of soil). The application of AgNO_3_ and polyethyleneimine AgNPs at 125 mg/kg of soil caused a significantly higher accumulation of silver in radish tissues (shoot, tuber and fine root) than other tested nanoparticles.

Matras et al. [38] compared the effects of silver ions from AgNO_3_ and various AgNPs of a similar size (positively charged CHSB-AgNPs prepared with cysteamine hydrochloride; negatively charged TCSB-AgNPs and TA-AgNPs prepared with trisodium citrate and tannic acid, respectively) on the germination and growth of monocots (wheat, sorghum (*Sorghum* Moench)) and dicots (cress, white mustard (*Sinapis alba* L.)). Each type of AgNP and Ag^+^ inhibited the growth of wheat and cress seedlings. The most toxic were positively charged nanoparticles. Moreover, researchers suggested that silver ions from AgNO_3_ were more toxic to monocots (wheat, sorghum), whereas positively charged silver nanoparticles were more toxic to dicots (cress, mustard) than negatively surface-charged AgNPs. It should be noted that the bio-AgNPs used in our study were negatively surface charged [16] and inhibited cress seedlings’ growth at each of the applied concentrations (Table S2), which is a new observation. Moreover, the phytotoxicity of bio-AgNPs on wheat seedlings (Figure S1B; Table S1) is a confirmation of our previous results in an experiment with another wheat cultivar [17].

Thus, the phytotoxicity or stimulatory effect of AgNPs on plants seems to be related to many factors, like their properties (i.e., size, shape, surface charge, and coating), excessive concentration, and plant species/developmental stage and/or organ. Another important factor is the level of released silver ions from AgNPs, as they are associated with nanoparticles’ toxicity. Many studies, including the present one, focus on the overall effect of nanoparticles, without distinguishing effects of nanoparticles and the ions released from them. Besides comparing the mechanism of Ag^+^ and AgNP action on plants, it is also important to analyze the level of ion release from nanoparticles depending on environmental conditions, as root exudates and soil leachate may influence the oxidative dissolution of nanoparticles and thus their stability and toxicity [39,40].

Although our results suggest that tested concentrations of bio-AgNPs were non-harmful to pea seeds’ germination (and subsequent seedlings’ development), their application to other species should be further investigated to elucidate the different levels of sensitivity of plants to the same nanoparticles.

### 3.2. Seedlings’ Viability, ROS Generation, and Antioxidant System

Live/dead fluorescent staining confirmed that bio-AgNPs did not affect the viability of seedlings’ root tips (Figure 1B). However, according to TTC staining, nanoparticles at the concentration of 200 mg/L affected cell respiration mostly in the root hair zone (Figure 2A,D). Moreover, bio-AgNPs at 50 mg/L stimulated the generation of ROS in root tips, which was not observed at a higher concentration (Figure 1A). In our previous study, where wheat seedlings were germinated for 3 days in bio-AgNPs at concentrations of 20–40 mg/L, an enhanced ROS level was reported in root tips [17]. However, such exposure of wheat seedlings also caused an increased number of dead cells in root tips, which was not observed in the present study for pea seedlings.

ROS play an important role as signaling molecules; thus, their content is tightly regulated by the balance between production and scavenging [41]. Specific ROS homeostasis is crucial for seed germination [42,43,44] and seedling growth [43,45], which confers cell division, elongation, and differentiation. Superoxide radicals and hydrogen peroxide are natural components of properly functioning cells. During the process of cellular respiration in mitochondria, O_2_^−^ radicals are formed in the electron transport chain and are then converted to H_2_O_2_ by superoxide dismutase (SOD) [46,47,48], and H_2_O_2_ is then decomposed by peroxidases [46]. In plants, superoxide radicals and hydrogen peroxide are also generated in the photosynthetic electron transport chain in chloroplasts [46,49]. Therefore, disturbance in reactive oxygen species generation might lead to disruption of antioxidative system homeostasis and thus proper cell functioning [50].

Nanoparticles’ toxicity is mostly associated with ROS generation and thus oxidative stress. It was reported that AgNPs can induce ROS generation in seedlings of turnip (*Brassica rapa* ssp. *rapa* L.) [51] and onion [20], as well as plants of lettuce (*Lactuca sativa* L.) [52] or pollen of kiwifruit (*Actinidia deliciosa* var. *deliciosa* (A. Chev) C. F. Liang et A. R. Ferguson) [53]. Pea seedling histochemical staining revealed that the presence of the O_2_^−^ in the roots was not affected by bio-AgNPs (Figure 2B,E), but the H_2_O_2_ level was greatly reduced (Figure 2C,F). It was reported that an increased concentration of AgNPs caused an increased superoxide ion content in pea, chickpea, and mung bean seedlings [13], which was not observed in the present study.

Low ROS occurrence in the root tips of seedlings developed in bio-AgNPs at the concentration of 200 mg/L (Figure 1A) might be partially explained by nanoparticles’ interaction with the antioxidant enzymes or their antioxidative properties [54,55]. The antioxidant properties of bio-AgNPs, described by Rilean-Plugaru et al. [16], expressed as the antiradical capacity, and thus the ability of nanoparticles to scavenge DPPH radicals, may be a result of the presence of metal silver nanoparticles and the organic coating, which was composed of amino acids like cysteine, isoleucine/leucine, serine, tryptophan, arginine, asparagine, phenylalanine, and tyrosine. Antioxidant properties were also presented for other silver nanoparticles. Moreover, biologically/green synthesized nanoparticles presented a higher antioxidant activity than those chemically synthesized, which is probably a result of the presence of the organic matter as a coating agent on the nanoparticle surface [54,55]. However, the AgNPs’ antioxidant properties are mainly associated with their interaction with the antioxidant enzymes, which enhance their activity [53]. Therefore, we also investigated the activity of selected antioxidative enzymes in pea seedlings developed in the presence of bio-AgNPs.

In our study, the main changes under bio-AgNP treatment were found in GPOX and APX activities (Table 2). Guaiacol peroxidase is associated with cytosol and cell walls, whereas ascorbate peroxidase in addition to that is also present in peroxisomes, mitochondria, and chloroplasts [55]. The reduced activity of mentioned peroxidases (especially in epicotyl; Table 2) may suggest damage to cells or cellular structures in which these enzymes occur, or transcription/translation disorders as AgNPs can exhibit cytotoxic and genotoxic effects [2,8,56]. However, no changes were observed in the length, FW, and DW of the epicotyl (Table 1). This with increased TAC in the epicotyl (Table 2) suggested the increased contribution of non-enzymatic antioxidants to maintain the redox balance [57].

Free radicals and peroxides interact with cell membranes, organelles, and genetic material, causing disruption of cell membranes, lipid peroxidation, and genotoxicity (chromosomal aberrations, cell division disorders) [8,50,58]. Therefore, to counteract oxidative stress induced by nanoparticles, plants enhance antioxidant enzyme activity [8,50,58]. However, changes in enzyme activity vary depending on the plant species and the type and concentration of nanoparticles. Daily application of AgNPs (biologically synthesized with *Pseudomonas plecoglossicida* post-culture medium) in the range of 20–100 mg/L stimulated the activity of CAT, APX, and SOD in 15-day-old pea seedlings [59]. A stimulatory effect of AgNPs on antioxidative enzymes was also present for lettuce, in both leaves and roots, especially after root exposure [52], and wheat plants [60]. Higher activity of GPOX was observed in tomato and kale seedlings after exposure to AgNPs (chemically synthesized) at a concentration of 100 mg/L, but decreased activity was observed in radish seedlings, whose root growth was stimulated by nanoparticles at this concentration. No changes in SOD activity were observed [25]. Such decreased activity of GPOX and no changes in SOD observed for radish seedlings resistant to AgNPs were similar to our findings for pea seedlings, which were also unaffected by bio-AgNPs.

Besides generating oxidative stress, AgNPs can also have direct toxic effects on plant cells. Nanoparticles can interact with various proteins, including antioxidative enzymes, stress-sensing proteins associated with the endoplasmic reticulum, membrane proteins, and other proteins involved in the proper functioning of cellular organelles, causing, e.g., disruption in the mitochondrial structure and fusion and fission, or ER homeostasis [8,50,58]. The present results also revealed that the tested concentrations of bio-AgNPs caused redox homeostasis alternation via the production of ROS (Figure 1A) and effect on antioxidative enzyme activity (Table 2), but did not exceed a certain threshold limit and thus did not injure pea seedlings (Table 1).

### 3.3. Bio-AgNPs Affected Metabolic Profiles of Pea Seedlings

The metabolic profile of 4-day-old pea seedlings was consistent with previous findings [14,61]. Germination in bio-AgNP suspension affected pea seedlings’ metabolism, even though no effect on pea seedlings’ growth/development was observed. However, seedlings’ metabolic rearrangements varied depending on the concentration of nanoparticles.

Sucrose (Suc) and raffinose family oligosaccharides (RFOs) are major soluble carbohydrates in the embryonic axis and cotyledons of mature pea seeds [61]. RFOs are hydrolyzed during the first few days of germination for energy purposes [62,63,64]. Thus, changes in the level of raffinose and stachyose in cotyledons suggest that bio-AgNPs at a concentration of 50 mg/L stimulated RFO hydrolysis, whereas at a higher concentration, such mobilization was greatly slowed down (Figure S3A; Table S5). Although AgNPs have been demonstrated to inhibit the activity of numerous enzymes (presumably as a result of the binding of the released Ag^+^ ions to the thiol groups of enzymes), among them, bacterial b-galactosidases [65], the inhibitory properties of bio-AgNPs against a-d-galactosidase, catalyzing the hydrolysis of RFOs remain to be explained.

Sucrose is the main transport form of sugars and can act as a signaling molecule [66,67,68]. Glucose and fructose serve as substrates for respiration and a source of carbon skeletons for other metabolic pathways [64]. Moreover, Suc and *myo*-inositol can act as osmoprotectants, stabilizing proteins or cell membranes during abiotic stresses [67,68,69]. Thus, an elevated level of sucrose in epicotyl (Figure 4A; Table S4) and cotyledons (Figure S3A; Table S5) and decreased level in root (without changes in the levels of fructose and glucose; Figure 4D; Table S3) of seedlings developing in bio-AgNPs at 50 mg/L suggest changes in Suc transport from storage tissues to growing tissues or that epicotyl was the preferable sink tissue. Indirect confirmation of this supposition can be found with elevated levels of monosaccharides and unchanged sucrose in the epicotyl of seedlings developed in bio-AgNPs at 200 mg/L. Moreover, the increased levels of monosaccharides in epicotyl were accompanied by lowered levels of citrate and malate in both epicotyl and root (Figure 4C,F), which may indicate changes in the tricarboxylic acid (TCA) cycle [70]. Moreover, glucose was not used to synthesize *myo*-inositol as its content decreased [69].

It was previously reported that AgNPs (20 mg/L; chemically synthesized) stimulated the accumulation of monosaccharides in the root and epicotyl of pea seedlings [14]. Foliar spray of wheat leaves with an AgNP (synthesized using sodium hexametaphosphate and sodium hypophosphite) suspension (at 10 mg/L) increased levels of kestose, sucrose, sorbitol, and raffinose, as well as positively affected yield parameters, such as the thousand-grain weight and the number of grains per spike [71].

The content of free amino acids did not change in the root and epicotyl of seedlings developed in bio-AgNPs (Tables S3 and S4), and thus their utilization for peptide and protein synthesis should not be affected [70,72]. However, in cotyledons, TAAs increased (Table S5), which suggested lower amino acid mobilization [70,72]. The observed rearrangements in the amino acid content included changes mainly in the concentrations of homoserine, glutamic acid, GABA, proline, and alanine (only in epicotyl). Homoserine is the most abundant non-proteinogenic amino acid in young pea seedlings [14,73,74,75]. Hse is synthesized from aspartate (Asp) and transported from the cotyledons to developing roots and epicotyls [73,75]. Hse is also utilized in the production of other amino acids such as threonine or methionine [74,76,77]. A higher concentration of homoserine in cotyledons accompanied by lower levels in both roots and epicotyls at a concentration of 50 mg/L of bio-AgNPs suggest disturbances in the synthesis of this amino acid (while indirectly in the mobilization of N from protein reserves). However, increased concentrations of lysine (Lys) in roots and epicotyls (Tables S3 and S4) and a higher concentration of threonine (Thr) in epicotyls (Table S4) suggest that the lower concentration of Hse may be caused partially by the switch in biosynthesis pathway from Asp→Hse to Asp→Lys and by the utilization of Hse for threonine synthesis. The increased use of homoserine for the synthesis of threonine was also confirmed by the greater amount of isoleucine (Ile; in epicotyls), as Thr is a precursor of Ile [77].

Some disturbances in the nitrogen reserves’ transport were also noted at the highest tested concentration of bio-AgNPs, due to the accumulation of Glu in cotyledons (Figure S2B; Table S5) and decrease in roots (Table S3), as Glu is one of the main form of N transport in plants, next to glutamine, asparagine, and aspartate [72,78,79]. Moreover, glutamate is also a precursor of many other amino acids, such as proline and GABA [72,80]. A lower concentration of bio-AgNPs stimulated an accumulation of proline in the pea root and epicotyl (Figure 4B,E; Tables S3 and S4), whereas the GABA content increased in roots (in epicotyl not significantly) at the highest bio-AgNP concentration (Tables S3 and S4). GABA is an important regulator of C: N metabolism via the GABA shunt. Glutamic acid is transported from the mitochondria to the cytoplasm and decarboxylated to GABA. Then, GABA is imported to the mitochondria and converted to succinic semialdehyde, and afterwards to succinic acid—an intermediate of the tricarboxylic acid (TCA) cycle. Another TCA intermediate, α-ketoglutarate, is converted to glutamate, and the GABA shunt can start again [81,82]. GABA as well as proline were reported to accumulate in plant tissues during various stresses. Both amino acids are considered as osmoprotectants and ROS scavengers [72,81,82,83,84,85]. Moreover, GABA is considered a more effective ROS quencher than Pro [86]. Proline can also act as a chemical chaperone and metal chelator [83,84], whereas GABA can stimulate the activity of antioxidative enzymes [85]. Thus, Pro and GABA might act as non-enzymatic ROS scavengers (stable TAC level; Table 1), but the stimulatory role of GABA to antioxidative enzymes is debatable as only APX activity increased in the roots (Table 1).

Alanine, the differentiating metabolite in epicotyl samples (Figure 3E), acts mainly as the precursor for the synthesis of other amino acids. It is also considered an osmolyte that maintains cellular integrity. Moreover, Ala is involved in nitrogen and, indirectly, carbon metabolism. It is synthesized by transamination of pyruvate, which is accompanied by the conversion of glutamate to α-ketoglutarate [87,88].

Accumulation of homoserine and a decreased level of proline were previously reported for pea seedlings developed in a chemically synthesized AgNP suspension at a concentration of 20 mg/L [14], and it was also observed in the present study for seedlings developed at 200 mg/L of bio-AgNPs. The main difference was observed for the TAA level, which was previously increased, suggesting that amino acids were not utilized for the synthesis of peptides and proteins [14], but no such observations were made in the present study. Amino acids’ decrease in content was also observed in Arabidopsis seedlings after soil application of AgNPs (12.5 mg/kg of soil), including increased levels of valine, serine, and aspartate and downregulation of tryptophan metabolism [89]. Also, foliar application of AgNPs (40 mg/plant) to cotton plants downregulated glutamine and asparagine synthesis in leaves, which suggested lower nitrogen fixation [90]. Thus, the unchanged level of amino acids suggests that bio-AgNPs did not disturb nitrogen metabolism but presumably triggered some rearrangements to maintain redox homeostasis (Pro, GABA), energetic resources (GABA), and cellular integrity (Ala). However, bio-AgNPs at a concentration of 200 mg/L appeared to impede nitrogen transport.

The elevated content of malic acid in the epicotyl of seedlings developed at 50 mg/L of bio-AgNPs (Table S4; Figure 4C) suggests increased cell respiration [68]. A low concentration of chemically synthesized AgNPs (20 mg/L) also stimulated the accumulation of malate and additionally citrate in the roots and epicotyls of pea seedlings [14]. An enhanced content of TCA cycle intermediates was also observed in Arabidopsis seedlings exposed to AgNPs [89] and cucumber leaves after AgNP foliar treatment [90]. However, malate and citrate significantly decreased in all analyzed tissues of seedings developed in the bio-AgNP suspension at a concentration of 200 mg/L (Figure 4C,F and Figure S2C; Tables S3–S5). Such a decrease in the levels of TCA intermediates indicates a decrease in respiration and lower energy supply [70]. Lower respiration in the roots was also confirmed by TTC staining (Figure 2A).

Bio-AgNPs at a concentration of 50 mg/L elevated the phosphoric acid content in all pea seedling tissues (Figure 4C,F and Figure S2C; Tables S3–S5). Our previous findings also showed elevated accumulation of phosphate in the roots and epicotyls of pea seedlings developed in AgNPs at 20 mg/L [14]. Phosphorus is an important constituent of phospholipids, nucleic acids, and proteins. Thus, it is crucial in various metabolic and physiological processes, including energy metabolism as a component of nucleotides, such as ATP [91,92]. The increased phosphoric acid content might be a result of increased ATP dephosphorylation. This, along with the increased malic acid content (Figure 4C,F), indicates increased demand for energy. At a higher concentration of bio-AgNPs, energetic demands of seedlings seemed to be stable, as the phosphoric acid levels in all tissues of pea seedlings were similar to the control (Figure 4C,F and Figure S2C; Tables S3–S5).

## 4. Materials and Methods

### 4.1. Biologically Synthesized Silver Nanoparticles

The biologically synthesized silver nanoparticles (bio-AgNPs) used in the present study were spherical in shape and homogeneous, with an average size of 18.3 ± 0.6 nm. The stability of the bio-AgNP dispersion was analyzed previously (for up to 6 days and it was reported that it was still stable [16]). According to the DLS method, just after dispersion preparation, 2 dominant particle populations were observed, ranging from 75 to 194 nm with a zeta potential value of −30 mV. The signal of the second population was more intense, which suggested that the predominantly hydrodynamic size of biocolloids is about 194 nm, and this particle size distribution remained constant for 3 days. Later, the size increased up to 490 nm and the zeta potential decreased to −25 mV. DLS and TEM results of bio-AgNP characterization indicate a stable colloidal system with a hydrodynamic diameter of 100 to 150 nm, a polydispersity index (PDI) below 0.7, and zeta potential values ranging from −23 to −41 mV, suggesting minimal aggregation and enhanced stability [16]. A detailed description of their biosynthesis, elemental composition, and physicochemical properties (surface composition, interactions between the metallic surfaces and organic ligands, and the content of elemental silver) was given earlier [16,22].

For the present experiments, an aqueous suspension of bio-AgNPs was prepared at concentrations of 50 and 200 mg/L in double-distilled water and sonicated 2 times for 30 min to obtain an appropriate nanoparticle distribution (Sonic-3, 310 W, 40 KHz, POLSONIC, Pałczyński, Poland).

### 4.2. Plant Material

Seeds of pea (*Pisum sativum* L.) cultivar Nemo (Danko Hodowla Roślin, Choryń, Poland), cv. Tarchalska (Danko Hodowla Roślin, Choryń, Poland), and cv. Sześciotygodniowy TOR (TORSEED—Przedsiębiorstwo Nasiennictwa Ogrodniczego i Szkółkarstwa S.A., Toruń, Poland), wheat (*Triticum aestivum* L.) cv. Ostka Smolicka (Hodowla Roślin Smolice, Smolice, Kobylin, Poland), radish (*Raphanus sativus* L.), and cress (*Lepidium sativum* L.) (PlantiCo—Hodowla i Nasiennictwo Ogrodnicze Zielonki, Stare Babice, Poland) were used to investigate their sensitivity to bio-AgNPs at concentrations of 50 and 200 mg/L.

Pea seeds were germinated for 4 days (22 °C, in the dark, in a climatic chamber ILW 115-T STD, Pol-Eko-Aparatura, Wodzisław Śląski, Poland) on Petri dishes (⌀ 120 mm) with water suspensions of bio-AgNPs at concentrations of 50 and 200 mg/L and water as a control. For each concentration of nanoparticles, we prepared 8 replicates with 20 seeds each for cv. Nemo and 3 replicates with 20 seeds each for cv. Tarchalska and Tor. After 4 days, seedlings from each replication were collected and measured.

Pea seedlings of cv. Nemo were selected for a more detailed analysis—the presence of reactive oxygen species, cell viability, antioxidant enzyme activity, total antioxidant capacity, and metabolic profiling. From each replication, a few seedlings were collected for microscopic analyses (root tips) and for histochemical staining (whole seedlings). The remaining pea seedlings were divided into epicotyls, roots, and cotyledons, weighed, and then frozen in liquid nitrogen and stored at −80 °C. Half of the samples of pea seedlings’ tissues were crushed in liquid nitrogen with a cold mortar and pestle for the determination of antioxidant enzyme activity and total antioxidative capacity, and the rest of them were lyophilized for metabolite profiling.

Seeds of wheat, radish, and cress were germinated for 4 days (22 °C, in the dark, in a climatic chamber ILW 115-T STD, Pol-Eko-Aparatura, Wodzisław Śląski, Poland) on Petri dishes (⌀ 120 mm for wheat, ⌀ 100 mm for radish and cress) with water suspensions of bio-AgNPs at concentrations of 50 and 200 mg/L and water as a control. There were 3 repetitions for each concentration, with at least 20 seeds each for wheat and radish and at least 30 seeds each for cress. After 4 days, properly developed seedlings from each replicate were measured and their fresh weight was determined. The different responses of radish and cress seeds to applied bio-AgNPs prompted us to conduct an additional test with a more precise spectrum of nanoparticle concentrations: 25, 50, 75, 100, 150, and 200 mg/L (for each concentration in 3 repetitions with 30 seeds each).

### 4.3. Presence of Reactive Oxygen Species and Cell Viability

#### 4.3.1. Fluorescence Staining

Reactive oxygen species (ROS) in root tips of pea seedlings incubated in bio-AgNPs at concentrations of 0, 50, and 200 mg/L were detected according to Benabdellah et al. [93]. Roots of pea seedlings were placed in 10 μM 2,7-dichlorodihydrofluorescein diacetate (H_2_DCF-DA; Sigma-Aldrich, St. Louis, MO, USA) in 0.1 M phosphate-buffered saline (PBS, pH = 7.4). After 30 min incubation in the dark, H_2_DCF-DA was replaced with fresh PBS for another 30 min and imaged by confocal laser scanning microscopy (CLSM; Leica TCS SP5, Leica Microsystems, Wetzlar, Germany) at an excitation wavelength λ_ex_ = 488 nm and emission wavelengths λ_em_ = 515–565 nm.

To identify cell viability within the root tip after incubation in bio-AgNPs, root tips of pea seedlings were stained with the mix of SYTO^®^ 9 and propidium iodide (PI) stain (LIVE/DEAD, L7007, Life Technologies, Carlsbad, CA, USA). After 15 min of incubation in the dark, samples were rinsed in water and imaged by CLSM at wavelengths for SYTO^®^ at 9 λ_ex_ = 488 nm and λ_em_ = 500–520 nm and for PI at λ_ex_ = 561 nm and λ_em_ = 610–650 nm. The PI solution was used to identify nonviable cells [94], and SYTO^®^ 9 was used to identify nonviable and viable cells [95].

#### 4.3.2. Histochemical Staining

The collected pea seedlings were gently rinsed with water to remove any remaining nanoparticles from the seedling surface. The following stains were used: staining with 2,3,5-triphenymtetrazolium chloride (TTC; Sigma-Aldrich, St. Louis, MO, USA) to determine cell viability, staining with 3,3-diaminobenzidine hydrochloride (DAB; Alfa Asear, Thermo Fisher, Kandel, Germany) to determine H_2_O_2_ localization, and staining with nitro blue tetrazolium chloride (NBT; Sigma-Aldrich, St. Louis, MO, USA) to determine localization of superoxide radicals (O_2_^−^).

TTC staining was performed according to Li et al. [96]. Seedlings were stained with 0.5% TTC solution at 35 °C for 1 h in the dark, then rinsed in distilled water and photographed. In living tissues, colorless TTC is reduced by dehydrogenases to red triphenylformazan [97].

DAB staining was performed according to Thordal-Christensen et al. [98]. Seedlings were stained with 1 mg/mL DAB-HCl (pH 3.8) for 90 min at room temperature in the dark, then rinsed in distilled water and photographed. DAB is oxidized by H_2_O_2_ in the presence of peroxidases and produces brown precipitate.

NBT staining was performed according to Zhang et al. [90]. Seedlings were stained with 1 mM NBT in 10 mM Tris-HCl buffer for 30 min at room temperature in the dark, then rinsed in distilled water and photographed. NBT reacts with O_2_^−^ to form a dark blue insoluble diformazan.

### 4.4. Antioxidant Enzyme Activity

Epicotyls, roots, and cotyledon samples (500 mg) were homogenized with a cold mortar and pestle in ice-cold 50 mM phosphate sodium buffer (pH 7.0) with 1 mM ethylenediaminetetraacetic acid (EDTA; AKTYN, Suchy Las, Poland), 1% polyvinylpyrrolidone (PVP; Sigma-Aldrich, St. Louis, MO, USA), and 0.1% Triton X-100 (AKTYN, Suchy Las, Poland). The samples were centrifuged at 20,000× *g* and 4 °C for 20 min. The supernatant was collected and stored on ice (4 °C). Prepared enzymatic extracts were used to spectrophotometrically (UV-1900i UV-Vis spectrophotometer, Shimadzu, Kyoto, Japan) determine the activity of catalase (CAT), ascorbate peroxidase (APX), guaiacol peroxidase (GPOX), and superoxide dismutase (SOD). The protein content of extracts was determined according to the Bradford assay [99], using bovine serum albumin (Fluka, Buchs, Switzerland) as the protein standard and Bradford reagent (Sigma-Aldrich, St. Louis, MO, USA).

CAT (EC 1.11.1.6) activity was performed according to Aebi [100]. The reaction mixture (final volume 2 mL) contained 50 mM phosphate sodium buffer (pH 7.0), 5 mM H_2_O_2_ (Tarchem, Góry Tarnowskie, Poland), and 200 µL of enzyme extract. Absorbance was measured for 5 min at room temperature and λ = 240 nm (ε = 36 M^−1^ cm^−1^). The CAT activity unit corresponds to the decomposition of 1 µmol of H_2_O_2_ for 1 min per 1 mg of protein.

APX (EC 1.11.1.11) activity was performed according to Nakano and Asada [101]. The reaction mixture (final volume 3 mL) contained 50 mM phosphate sodium buffer (pH 7.0), 0.25 mM ascorbic acid (Stanlab, Lublin, Poland), 1 mM H_2_O_2_, and 100 µL of enzyme extract. Absorbance was measured for 5 min at room temperature and λ = 290 nm (ε = 2.8 mM^−1^ cm^−1^). The APX activity unit corresponds to the oxidation of 1 µmol of ascorbate for 1 min per 1 mg of protein.

GPOX (EC 1.11.1.7) activity was performed according to Mika and Lüthje [102]. The reaction mixture (final volume 3 mL) contained 50 mM phosphate sodium buffer (pH 7.0), 0.3 mM guaiacol (Sigma-Aldrich, St. Louis, MO, USA), 3 mM H_2_O_2_, and 150 µL of enzyme extract. Absorbance was measured for 10 min at room temperature and λ = 470 nm (ε = 26.6 mM^−1^ cm^−1^). The GPOX activity unit corresponds to the oxidation of 1 µmol of H_2_O_2_ for 1 min per 1 mg of protein.

SOD (EC 1.15.1.1) activity was performed according to Beauchamp and Fridovich [103]. The reaction mixture (final volume 3 mL) contained 50 mM phosphate sodium buffer (pH 7.0), 75 µM nitro blue tetrazolium (NBT; Sigma-Aldrich, St. Louis, MO, USA), 20 mM methionine (Sigma-Aldrich, St. Louis, MO, USA), 0.1 mM EDTA, 0.1% Triton X-100, 2 µM riboflavin (Sigma-Aldrich, St. Louis, MO, USA), and 150 µL of enzyme extract. The reaction mixture with phosphate buffer instead of enzyme extract was used as a control sample, where the maximum reduction in NBT by O_2_^−^ occurred. Samples and the control were incubated under light (2 × 8 W) for 10 min. Unilluminated samples were used as a blank. Absorbance was measured at λ = 560 nm. The SOD activity unit (U) corresponds to an inhibition by 50% of the reduction in NBT in comparison to the control per 1 mg of protein.

### 4.5. Total Antioxidant Capacity

The total antioxidant capacity (TAC) was determined based on the modified method of Michalska et al. [104], using radicals of 2,2-diphenyl-1-picrylhydrazyl (DPPH^•^), which were reduced by antioxidants present in the sample. Samples of pea seedlings’ tissues (500 mg) were extracted with 80%(*v*/*v*) methanol for 2 h at room temperature with continuous shaking. The samples were centrifuged at 10,000× *g* and 4 °C for 15 min. The supernatant was collected and stored on ice (4 °C). The reaction mixture contained 1.6 mL of 80%(*v*/*v*) methanol, 125 µL of DPPH^•^ (Sigma-Aldrich, St. Louis, MO, USA), and 100 µL of sample methanolic extract or 100 µL of 80% methanol for a blank. Vortexed samples were incubated in the dark at room temperature for 30 min. Absorbance was measured at λ = 517 nm using a UV-1900i spectrophotometer (Shimadzu, Japan). Trolox (Thermo Scientific, Waltham, MA, USA) was used to prepare a standard curve (range from 0 to 500 µM in 80%(*v*/*v*) methanol), assayed in the same conditions. The total antioxidant capacity of samples was expressed as the Trolox equivalent antioxidant capacity (TEAC), based on the Trolox standard curve, per g of FW.

### 4.6. Metabolite Profiling

Lyophilized and pulverized (mixer mill MM200, Retsch, Haan, Germany) pea seedling tissue samples were subjected to polar metabolite analysis according to Szablińska-Piernik and Lahuta [105]. Briefly, polar metabolites were extracted using the mixture of methanol and water (1:1, *v*:*v*) at 70 °C for 30 min with continuous shaking. Ribitol was used as the internal standard (1 mg/mL). Then, the homogenates were cooled on ice and centrifuged (20,000× *g* at 4 °C for 20 min), and the supernatants were mixed with cold chloroform to remove non-polar compounds. Dried samples were derivatized with *O*-methoxamine hydrochloride and a mixture of *N*-methyl-*N*-trimethylsilyl-trifluoroacetamide (MSTFA) with pyridine (1:1, *v*/*v*). The trimethylsilyl (TMSI) derivatives were separated on a capillary column ZEBRON ZB-5MSi Guardian (length 30 m, diameter 0.25 mm, film 0.25 μm; Phenomenex, Torrance, CA, USA) in the gas chromatograph GC2010 Nexia (Shimadzu, Kyoto, Japan) with a flame ionization detector (FID). Metabolites were identified and characterized by the comparison of their retention times (RTs), retention indices (RIs, determined according to the saturated hydrocarbons), and mass spectra of original standards derived from Sigma-Aldrich (Sigma-Aldrich, Merck, Burlington, MA, USA). Additionally, the same samples were also separated in the gas chromatograph coupled with mass spectrometry (QP-GC-2010, Shimadzu, Japan) to confirm there was accurate metabolite identification, according to the NIST 05 library (National Institute of Standards and Technology, Gaithersburg, MD, USA).

### 4.7. Statistical Analyses

The results are the means of at least 3 independent replicates, and they were subjected to one-way ANOVA with a post hoc test (Tukey, if overall *p* ≤ 0.05) using Statistica software (version 12.0; StatSoft, Tulsa, OK, USA). Graphs were prepared using GraphPad Prism, version 8 (GraphPad Software, San Diego, CA, USA). Multivariate statistics of metabolomic data were analyzed using principal component analysis (PCA) and performed using COVAIN [106], a MATLAB toolbox including a graphical user interface (MATLAB version 2013a; Math Works, Natick, MA, USA).

## 5. Conclusions

Global climate changes and natural resource depletion pose a serious threat to agriculture and thus food and economic security. Moreover, intensive application of fertilizers and pesticides causes contamination of the environment. The use of nanoparticles in agricultural production (as seed-priming agents) seems to be an interesting solution that could reduce the applied amounts of pesticides while increasing yield quantity and quality. However, a fuller understanding of how nanoparticles affect physiological and biochemical reactions, and which ones are key to developing a safe and sustainable strategy for their use in agriculture, is needed. Therefore, in this study, we focused on the effect of bio-AgNPs, previously concluded to have potential as antifungal agents, on the metabolic rearrangements during pea seedling early development.

In the present study, no harmful effect of bio-AgNPs (50 and 200 mg/L) on pea seeds’ germination and seedlings’ growth were observed. Pea seedling adjustment to bio-AgNPs was related to changes in antioxidative enzymes’ activity and seedlings’ metabolic profiles.

Nanoparticles at both tested concentrations did not negatively affect root tips cells’ viability, but a lower concentration of bio-AgNPs stimulated ROS generation. Moreover, bio-AgNPs increased the activity of ascorbate peroxidase in the root, which resulted in a lower H_2_O_2_ level. The increased TAC in epicotyls might suggest the increased contribution of non-enzymatic antioxidants to maintain the redox balance, which is also supported by the increased levels of proline and GABA. Bio-AgNPs at a concentration of 50 mg/L exhibited a more stimulatory effect—increased levels of citrate, malate, phosphoric acid, and GABA indicate enhanced cellular respiration and energy production. Moreover, increased levels of alanine, proline, sucrose, and *myo*-inositol might be a result of rearrangement to maintain cellular integrity and osmotic stability. However, a higher concentration of bio-AgNPs seemed to impede nitrogen transport, but the energetic demands of seedlings seemed stable, at a level similar to control seedlings.

The presented results showed that bio-AgNPs altered redox homeostasis but did not exceed a certain threshold limit and thus did not injure pea seedlings, because of seedling metabolic adjustment. Nonetheless, the mechanism of pea seedlings’ resistance to bio-AgNPs remains unresolved and further investigations are necessary.

Moreover, the toxicity of bio-AgNPs is species-specific. No harmful effects were observed for pea and radish seeds’ germination and seedlings’ development, but wheat and cress seedlings’ growth were significantly decreased. Therefore, bio-AgNPs’ application to other species should be further investigated to understand the reasons for the different degrees of sensitivity of plants to the same nanoparticles.

## Figures and Tables

**Table 1:** Length, fresh weight (FW), dry weight (DW) of epicotyl (E), root (R), and cotyledon (C) of 4-day-old pea (*Pisum sativum* L.) seedlings developed in suspensions of bio-AgNPs at concentrations of 0, 50, and 200 mg/L. Means of 3 replicates ± SD. The same letters indicate statistically insignificant (*p* ≤ 0.05) differences (valid separately for data in rows) based on ANOVA and Tukey’s post hoc test.

		bio-AgNPs		
		0 mg/L	50 mg/L	200 mg/L
Length (mm)	R	24.2 ± 3.5 ^a^	28.2 ± 2.01 ^a^	27.9 ± 1.8 ^a^
	E	9.8 ± 1.4 ^a^	9.0 ± 1.1 ^a^	9.5 ± 0.8 ^a^
Fresh weight (mg)	R	43.8 ± 2.8 ^a^	45.2 ± 4.8 ^a^	48.5 ± 4.0 ^a^
	E	36.2 ± 5.4 ^a^	37.5 ± 2.4 ^a^	37.6 ± 2.1 ^a^
	C	279.3 ± 8.4 ^a^	291.6 ± 6.9 ^a^	283.9 ± 9.7 ^a^
Dry weight (mg)	R	3.5 ± 0.4 ^a^	3.9 ± 0.6 ^a^	4.4 ± 0.4 ^a^
	E	3.7 ± 0.4 ^a^	3.7 ± 0.3 ^a^	3.9 ± 0.5 ^a^
	C	123.2 ± 0.9 ^a^	123.6 ± 1.3 ^a^	113.9 ± 3.6 ^b^
Germinability (%)		92.2 ± 6.2 ^a^	93.6 ± 5.6 ^a^	95.6 ± 3.2 ^a^

**Table 2:** Activity of selected antioxidative enzymes and total antioxidant capacity (TAC) in epicotyl, root, and cotyledons of 4-day-old pea (*Pisum sativum* L.) seedlings developed in suspensions of bio-AgNPs at 0, 50, and 200 mg/L. Values (in mg/g DW) are means of 3 replicates ± SD. The same letters by the values indicate no statistically significant (*p* ≤ 0.05) differences (valid separately for data in rows) based on ANOVA and Tukey’s post hoc test.

		bio-AgNPs		
		0 mg/L	50 mg/L	200 mg/L
CAT (µmol H_2_O_2_ min^−1^ mg^−1^ protein)	Root	9.61 ± 0.34 ^a^	11.29 ± 1.58 ^a^	10.49 ± 0.33 ^a^
	Epicotyl	10.16 ± 1.79 ^a^	8.58 ± 0.11 ^a^	9.14 ± 0.77 ^a^
	Cotyledons	2.79 ± 0.18 ^a^	1.98 ± 0.06 ^b^	2.03 ± 0.17 ^b^
APX (µmol ASC min^−1^ mg^−1^ protein)	Root	0.16 ± 0.00 ^b^	0.19 ± 0.00 ^a^	0.19 ± 0.01 ^a^
	Epicotyl	0.21 ± 0.00 ^a^	0.18 ± 0.00 ^b^	0.19 ± 0.00 ^b^
	Cotyledons	0.02 ± 0.00 ^a^	0.02 ± 0.00 ^a^	0.02 ± 0.00 ^a^
GPOX (µmol H_2_O_2_ min^−1^ mg^−1^ protein)	Root	0.15 ± 0.01 ^a^	0.15 ± 0.00 ^a^	0.16 ± 0.01 ^b^
	Epicotyl	0.29 ± 0.00 ^a^	0.27 ± 0.00 ^c^	0.28 ± 0.00 ^b^
	Cotyledons	0.02 ± 0.00 ^a^	0.01 ± 0.00 ^b^	0.01 ± 0.00 ^ab^
SOD (U mg^−1^ protein)	Root	11.33 ± 0.56 ^a^	12.90 ± 0.67 ^a^	11.57 ± 0.70 ^a^
	Epicotyl	9.68 ± 1.07 ^a^	9.59 ± 0.18 ^a^	10.39 ± 0.16 ^a^
	Cotyledons	2.25 ± 0.09 ^b^	2.54 ± 0.09 ^a^	2.20 ± 0.11 ^b^
TAC (µM TEAC g^−1^ FW)	Root	1.58 ± 0.06 ^a^	1.68 ± 0.04 ^a^	1.60 ± 0.06 ^a^
	Epicotyl	2.11 ± 0.02 ^b^	2.21 ± 0.05 ^a^	2.20 ± 0.10 ^ab^
	Cotyledons	1.55 ± 0.09 ^a^	1.74 ± 0.07 ^a^	1.82 ± 0.11 ^a^

CAT—catalase; APX—ascorbate peroxidase; GPOX—guaiacol peroxidase; SOD—superoxide dismutase; TAC—total antioxidant capacity; TEAC—Trolox equivalent antioxidative capacity.

## Data Availability

The original contributions presented in this study are included in the article and Supplementary Materials. Further inquiries can be directed to the corresponding authors.

## Supplementary Materials

The following supporting information can be downloaded at https://www.mdpi.com/article/10.3390/plants14111594/s1, Figure S1: The 4-day-old seedlings of (A) pea (*Pisum sativum* L.), (B) wheat (*Triticum aestivum* L.), (C) radish (*Raphanus sativus* L.), and (D) cress (*Lepidium sativum* L.) developed in suspension of bio-AgNPs at concentrations of 0, 50, and 200 mg/L; Table S1. Fresh weight (FW) and length of 4-day-old seedlings of pea (*Pisum sativum* L.) cv. Tarchalska and cv. Sześciotygodniowy and wheat (*Triticum aestivum* L. cv. Ostka Smolicka) developed in suspension of bio-AgNPs at concentrations of 0, 50, and 200 mg/L; Table S2: Seedlings’ fresh weight (FW) and length of root and coleoptile/hypocotyl of 4-day-old seedlings of radish (*Raphanus sativus* L.) and cress (*Lepidium sativum* L.) developed in suspension of bio-AgNPs at concentrations of 0, 25, 50, 75, 100, 150, and 200 mg/L; Figure S2: The concentrations of selected metabolites in the cotyledons (A–C) of 4-day-old pea seedlings developed in suspension of bio-AgNPs at concentrations of 0, 50, and 200 mg/L; Tables S3–S5: The concentration of total identified polar metabolites (TIPMs), including total soluble carbohydrates (TSCs), total amino acids (TAAs), total organic acids (TOAs), and total remaining compounds (TRCs) in in shoots, roots, and cotyledons, respectively, of 4-day-old seedlings of pea (*Pisum sativum* L.) cv. Nemo developed in water suspensions of bio-AgNPs at a concentration of 0, 50, and 200 mg/L.

## Abbreviations

The following abbreviations are used in this manuscript:

bio-AgNPs	Biologically synthesized silver nanoparticles
NPs	Nanoparticles
ROS	Reactive oxygen species
CAT	Catalase
APX	Ascorbate peroxidase
GPOX	Guaiacol peroxidase
SOD	Superoxide dismutase
TEAC	Trolox equivalent antioxidant capacity
TAC	Total antioxidant capacity
PCA	Principal component analysis
TIPMs	Total identified polar metabolites
TSCs	Total soluble carbohydrates
TAAs	Total amino acids
TOAs	Total organic acids
TRCs	Total remaining compounds
