# Supplementary material for: The Resistance of Germinating Pea (Pisum sativum L.) Seeds to Silver Nanoparticles
Source: Plants (Basel). 2025 May 23;14(11):1594. doi: 10.3390/plants14111594 (PMC12157791; doi:10.3390/plants14111594)
Supplement: Supplementary file 1 [file plants-14-01594-s001.zip › plants-3615483-supplementary.pdf]

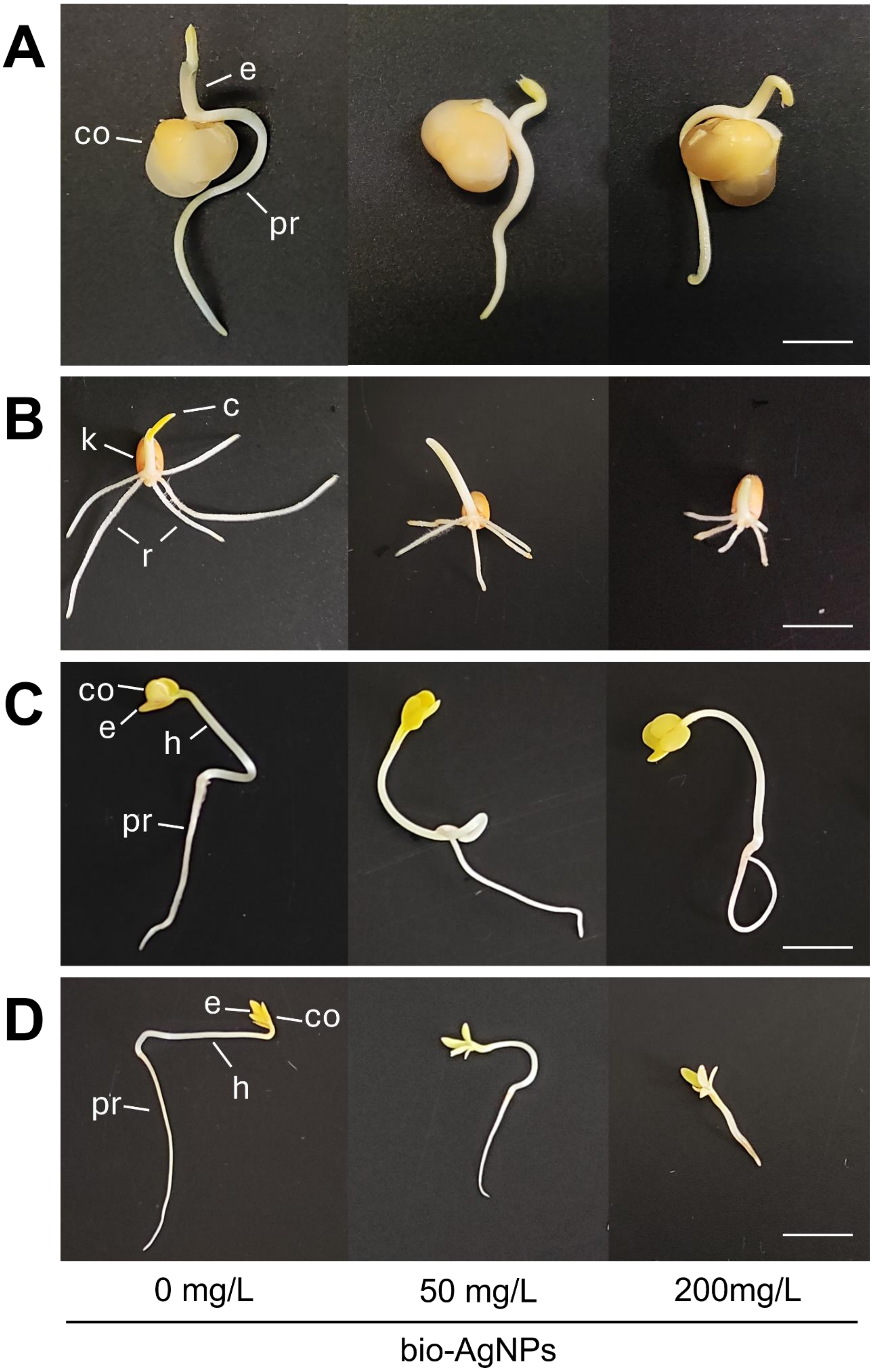

Figure S1. The 4-day-old seedlings of (**A**) pea (*Pisum sativum* L.), (**B**) wheat (*Triticum aestivum* L.), (**C**) radish (*Raphanus sativus* L.) and (**D**) cress (*Lepidium sativum* L.) developed in suspension of bio-AgNPs at concentrations of 0, 50 and 200 mg/L. Horizontal scale bars equal 10 mm. Abbreviations: c – coleoptile; e – epicotyl; h – hypocotyl; r – root; pr – primary root; co – cotyledons; k – kernel.

Table S1. Fresh weight (FW) and length of 4-day-old seedlings of pea (*Pisum sativum* L.) cv. Tarchalska and cv. Sześciotygodniowy and wheat (*Triticum aestivum* L. cv. Ostka Smolicka) developed in suspension of bio-AgNPs at concentrations of 0, 50 and 200 mg/L. Means of 3 replicates ± SD. The same letters indicate statistically insignificant ($p \leq 0.05$) differences (valid separately for data in rows) based on ANOVA analysis and Tukey's post-hoc test.

			**bio-AgNPs**		
			**0 mg/L**	**50 mg/L**	**200 mg/L**
Pea (*Pisum sativum* L.) cv. Tarchalska	FW (mg)	Root	37.1 ± 2.4 [a]	59.3 ± 25.2 [a]	47.7 ± 12.6 [a]
		Epicotyl	34.1 ± 0.6 [a]	37.8 ± 0.7 [a]	36.0 ± 2.6 [a]
		Cotyledons	242.6 ± 5.9 [a]	259.1 ± 14.6 [a]	251.1 ± 18.0 [a]
	Length (mm)	Root	23.2 ± 0.6 [a]	27.6 ± 3.9 [a]	31.1 ± 5.1 [a]
		Epicotyl	5.5 ± 0.0 [a]	6.9 ± 1.0 [a]	7.1 ± 1.5 [a]
Pea (*Pisum sativum* L.) cv. Sześciotygodniowy	FW (mg)	Root	49.3 ± 13.7 [a]	58.1 ± 22.4 [a]	52.1 ± 14.1 [a]
		Epicotyl	43.1 ± 4.8 [a]	35.1 ± 7.2 [a]	40.0 ± 4.7 [a]
		Cotyledons	224.8 ± 21.0 [a]	232.7 ± 27.8 [a]	231.0 ± 7.6 [a]
	Length (mm)	Root	25.4 ± 3.0 [a]	31.5 ± 10.9 [a]	33.9 ± 5.0 [a]
		Epicotyl	11.1 ± 3.6 [a]	10.3 ± 4.5 [a]	10.0 ± 2.1 [a]
Wheat *Triticum aestivum* L. cv. Ostka Smolicka	FW (mg)	Seedling	43.7 ± 4.8 [a]	36.7[ab] ± 2.4	33.6[b] ± 2.5
	Length (mm)	Root*	26.6 ± 5.0 [a]	17.1 ± 1.7 [b]	13.4 ± 0.6 [b]
		Coleoptile	13.3 ± 2.0 [a]	11.8 ± 1.4 [a]	9.5 ± 1.5 [a]

* - length of the primary root

Table S2. Seedlings' fresh weight (FW) and length of root and coleoptile/hypocotyl of 4-day-old seedlings of radish (*Raphanus sativus* L.) and cress (*Lepidium sativum* L.) developed in suspension of bio-AgNPs at concentrations of 0, 25, 50, 75, 100, 150 and 200 mg/L. Means of 3 replicates ± SD. The same letters indicate statistically insignificant ($p \leq 0.05$) differences (valid separately for data in columns and separately for each species) based on ANOVA analysis and Tukey's post-hoc test.

		FW (mg)	Length (mm)		
	**bio-AgNPs concentration (mg/L)**	Seedling	Root	Hypocotyl	Root + Hypocotyl
Radish *Raphanus sativus* L.	0	66.0 ± 8.7 [ab]	36.9 ± 3.6 [bc]	23.8 ± 1.4 [b]	60.7 ± 2.8 [bc]
	25	76.9 ± 16.5 [ab]	40.4 ± 2.7 [abc]	28.0 ± 2.2 [ab]	68.5 ± 4.9 [abc]
	50	83.7 ± 7.2 [a]	37.5 ± 6.2 [bc]	31.5 ± 3.4 [a]	69.0 ± 9.1 [abc]
	75	88.8 ± 13.4 [a]	49.5 ± 3.6 [a]	30.8 ±1.3 [ab]	80.3 ± 4.9 [a]
	100	84.8 ± 8.0 [a]	41.5 ± 4.4 [ab]	32.3 ± 2.0 [a]	73.8 ± 6.3 [ab]
	150	68.0 ± 7.7 [ab]	29.5 ± 2.5 [c]	28.0 ± 4.5 [ab]	57.5 ± 5.2 [c]
	200	57.5 ± 5.8 [b]	17.6 ± 0.3 [d]	13.6 ± 1.0 [c]	31.2 ± 1.3 [d]
Cress *Lepidium sativum* L.	0	25.6 ± 0.6 [a]	31.3 ± 2.7 [a]	23.2 ± 2.6 [a]	54.5 ± 1.5 [a]
	25	23.0 ± 3.9 [ab]	26.5 ± 4.1 [ab]	17.9 ± 3.4 [abc]	44.4 ± 7.4 [ab]
	50	21.7 ± 2.0 [ab]	23.2 ± 2.3 [abc]	19.1 ± 2.5 [ab]	42.3 ± 4.6 [ab]
	75	18.2 ± 1.1 [abc]	24.2 ± 4.6 [ab]	15.8 ± 0.7 [bcd]	39.9 ± 1.3 [abc]
	100	19.3 ± 2.2 [abc]	19.5 ± 6.0 [bcd]	16.4 ± 2.6 [bcd]	36.0 ± 8.6 [bcd]
	150	16.4 ± 5.4 [bc]	13.5 ± 2.1 [cd]	12.2 ± 0.9 [cd]	25.7 ± 2.9 [cd]
	200	12.3 ± 3.4 [c]	12.3 ± 3.3 [d]	11.2 ± 1.5 [d]	23.6 ± 4.8 [d]

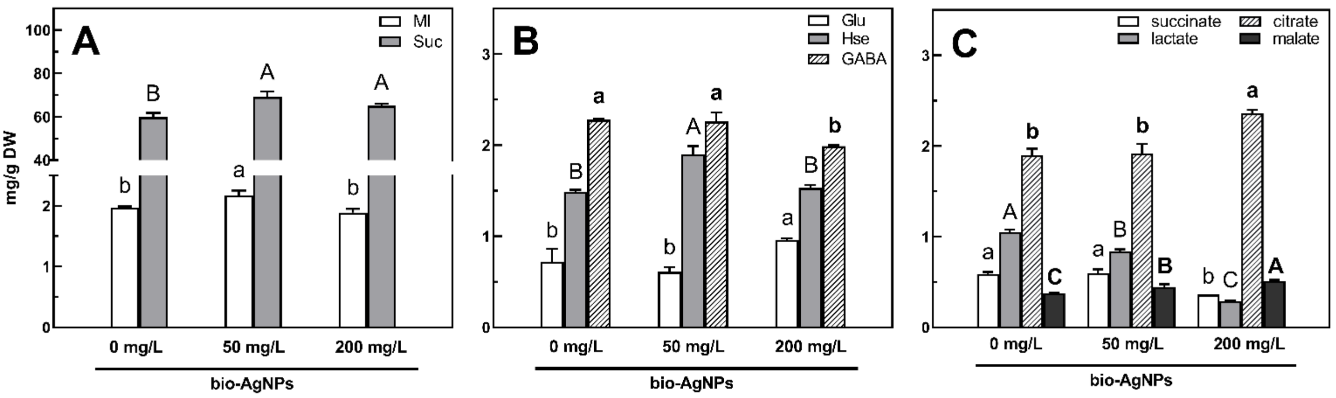

Figure S2. The concentrations of selected metabolites in the cotyledons (**A–C**) of 4-days-old pea (*Pisum sativum* L.) seedlings developed in suspension of bio-AgNPs at concentrations of 0, 50 and 200 mg/L. Values (in mg/g DW) are the means of 3 replicates + SD. The same letters (a–d; A–D; **a–c**; **A–D**; separately for each metabolite) above the bars indicate no statistically significant ($p \leq 0.05$) differences based on ANOVA and Tukey's post–hoc test. Abbreviations: MI – *myo*-inositol; Suc – sucrose; Glu – glutamic acid; Hse – homoserine; GABA – γ-aminobutyric acid.

Table S3. The concentration of total identified polar metabolites (**TIPMs**), including total soluble carbohydrates (**TSCs**), total amino acids (**TAAs**), total organic acids (**TOAs**), and total remaining compounds (**TRCs**) in **roots** of 4-day-old seedlings of pea (*Pisum sativum* L.) cv. Nemo developed in water suspensions of bio-AgNPs at a concentration of 0, 50 and 200 mg/L. Values (in mg/g DW) are means of 3 replicates ± SD. The same letters by the values indicate no statistically significant ($p \leq 0.05$) differences (valid separately for data in rows) based on ANOVA analysis and Tukey's post-hoc test.

	**bio-AgNPs**		
	**0 mg/L**	**50 mg/L**	**200 mg/L**
**Metabolites**	mg/g DW		
**TIPMs**, including:	**134.33 ± 2.30 ^{a}**	**123.99 ± 2.66 ^{b}**	**124.01 ± 3.23 ^{b}**
**TSCs**, including:	56.05 ± 1.18 ^{a}	47.10 ± 1.26 ^{c}	52.65 ± 1.09 ^{b}
fructose	0.48 ± 0.09 ^{a}	0.56 ± 0.09 ^{a}	0.53 ± 0.02 ^{a}
galactose	1.07 ± 0.10 ^{a}	0.88 ± 0.04 ^{b}	1.02 ± 0.04 ^{ab}
glucose	2.00 ± 0.29 ^{a}	1.92 ± 0.65 ^{a}	1.78 ± 0.08 ^{a}
myo-inositol	2.81 ± 0.12 ^{a}	2.65 ± 0.12 ^{a}	2.18 ± 0.07 ^{b}
sucrose	49.29 ± 0.81 ^{a}	40.68 ± 0.35 ^{c}	46.87 ± 1.08 ^{b}
gluconic acid	0.39 ± 0.03 ^{a}	0.42 ± 0.02 ^{a}	0.27 ± 0.01 ^{b}
**TAAs**, including:	53.04 ± 2.69 ^{a}	51.12 ± 1.04 ^{a}	50.37 ± 1.62 ^{a}
alanine	6.87 ± 0.47 ^{a}	7.35 ± 0.48 ^{a}	7.10 ± 0.43 ^{a}
asparagine	2.60 ± 1.14 ^{a}	2.74 ± 0.65 ^{a}	2.60 ± 0.07 ^{a}
aspartic acid	1.21 ± 0.08 ^{a}	1.11 ± 0.05 ^{ab}	1.02 ± 0.05 ^{b}
β-alanine	0.34 ± 0.29 ^{a}	0.37 ± 0.20 ^{a}	0.49 ± 0.03 ^{a}
GABA	1.39 ± 0.12 ^{b}	1.43 ± 0.13 ^{ab}	1.67 ± 0.04 ^{a}
glutamic acid	2.25 ± 0.11 ^{a}	2.31 ± 0.07 ^{a}	1.97 ± 0.07 ^{b}
homoserine	22.49 ± 0.13 ^{a}	19.04 ± 0.23 ^{c}	21.61 ± 0.54 ^{b}
hydroxyproline	1.84 ± 0.25 ^{a}	2.04 ± 0.25 ^{a}	1.73 ± 0.05 ^{a}
isoleucine	1.40 ± 0.03 ^{a}	1.42 ± 0.02 ^{a}	1.17 ± 0.06 ^{b}
leucine	0.18 ± 0.02 ^{b}	0.22 ± 0.02 ^{ab}	0.24 ± 0.01 ^{a}
lysine	1.33 ± 0.20 ^{ab}	1.62 ± 0.08 ^{a}	1.21 ± 0.06 ^{b}
phenylalanine	1.74 ± 0.22 ^{a}	1.68 ± 0.05 ^{a}	1.47 ± 0.05 ^{a}
proline	3.00 ± 0.08 ^{b}	3.19 ± 0.04 ^{a}	1.97 ± 0.05 ^{c}
serine	2.95 ± 0.11 ^{a}	2.75 ± 0.07 ^{a}	2.81 ± 0.08 ^{a}
threonine	0.57 ± 0.09 ^{a}	0.56 ± 0.06 ^{a}	0.53 ± 0.03 ^{a}
tyrosine	0.65 ± 0.07 ^{b}	0.95 ± 0.02 ^{a}	0.66 ± 0.02 ^{b}
valine	2.21 ± 0.10 ^{ab}	2.34 ± 0.03 ^{a}	2.12 ± 0.09 ^{b}
**TOAs**, including:	13.15 ± 0.40 ^{a}	13.14 ± 0.18 ^{a}	9.58 ± 0.22 ^{b}
citric acid	3.12 ± 0.25 ^{a}	3.17 ± 0.11 ^{a}	1.92 ± 0.04 ^{b}
fumaric acid	0.23 ± 0.02 ^{a}	0.22 ± 0.01 ^{a}	0.17 ± 0.02 ^{b}
lactic acid	0.07 ± 0.01 ^{a}	0.07 ± 0.02 ^{a}	0.06 ± 0.00 ^{a}
malic acid	8.89 ± 0.17 ^{a}	8.74 ± 0.18 ^{a}	6.51 ± 0.17 ^{b}
malonic acid	0.06 ± 0.00 ^{b}	0.07 ± 0.00 ^{a}	0.04 ± 0.00 ^{c}
oxalic acid	0.17 ± 0.03 ^{a}	0.20 ± 0.07 ^{a}	0.14 ± 0.00 ^{a}
propionic acid	0.12 ± 0.01 ^{a}	0.09 ± 0.01 ^{b}	0.10 ± 0.00 ^{ab}
succinic acid	0.50 ± 0.13 ^{a}	0.57 ± 0.11 ^{a}	0.64 ± 0.02 ^{a}
**TRCs**, including:	12.09 ± 0.27 ^{ab}	12.63 ± 0.26 ^{a}	11.41 ± 0.32 ^{b}
phosphoric acid	12.01 ± 0.26 ^{ab}	12.54 ± 0.26 ^{a}	11.31 ± 0.32 ^{b}
urea	0.08 ± 0.02 ^{a}	0.09 ± 0.03 ^{a}	0.10 ± 0.01 ^{a}

Table S4. The concentration of total identified polar metabolites (**TIPMs**), including total soluble carbohydrates (**TSCs**), total amino acids (**TAAs**), total organic acids (**TOAs**), and total remaining compounds (**TRCs**) in **epicotyls** of 4-day-old seedlings of pea (*Pisum sativum* L.) cv. Nemo developed in water suspensions of bio-AgNPs at a concentration of 0, 50 and 200 mg/L. Values (in mg/g DW) are means of 3 replicates ± SD. The same letters by the values indicate no statistically significant ($p \leq 0.05$) differences (valid separately for data in rows) based on ANOVA analysis and Tukey's post-hoc test.

	**bio-AgNPs**		
	**0 mg/L**	**50 mg/L**	**200 mg/L**
**Metabolites**	mg/g DW		
**TIPMs**, including:	**142.84 ± 1.28** [b]	**153.66 ± 4.97** [a]	**149.86 ± 2.56** [ab]
**TSCs**, including:	73.23 ± 0.29 [b]	80.53 ± 2.72 [a]	81.25 ±1.99 [a]
fructose	0.79 ± 0.06 [b]	0.66 ± 0.05 [c]	2.20 ± 0.04 [a]
galactose	0.41 ± 0.01 [b]	0.38 ± 0.01 [b]	1.91 ± 0.22 [a]
glucose	0.55 ± 0.04 [b]	0.66 ± 0.10 [b]	5.48 ± 0.25 [a]
myo-inositol	3.66 ± 0.22 [a]	3.79 ± 0.12 [a]	2.81 ± 0.15 [b]
sucrose	67.18 ± 0.33 [b]	74.36 ± 2.49 [a]	68.22 ± 1.97 [b]
gluconic acid	0.64 ± 0.02 [a]	0.67 ± 0.05 [a]	0.62 ± 0.02 [a]
**TAAs**, including:	50.04 ± 1.05 [a]	51.74 ± 1.55 [a]	50.46 ± 0.20 [a]
alanine	6.14 ± 0.15 [c]	8.86 ± 0.10 [a]	6.62 ± 0.16 [b]
asparagine	2.83 ± 0.05 [a]	3.23 ± 0.15 [a]	3.25 ± 0.28 [a]
aspartic acid	1.33 ± 0.01 [a]	1.21 ± 0.07 [a]	1.03 ± 0.05 [b]
β-alanine	0.52 ± 0.02 [a]	0.52 ± 0.02 [a]	0.48 ± 0.00 [a]
GABA	3.53 ± 0.15 [a]	3.57 ± 0.15 [a]	3.79 ± 0.10 [a]
glutamic acid	1.58 ± 0.05 [b]	1.99 ± 0.16 [a]	1.27 ± 0.05 [c]
homoserine	20.27 ± 0.51 [a]	16.22 ± 0.49 [b]	19.41 ± 0.34 [a]
hydroxyproline	1.32 ± 0.03 [b]	1.57 ± 0.05 [b]	1.91 ± 0.21 [a]
isoleucine	1.39 ± 0.04 [b]	1.54 ± 0.06 [a]	1.39 ± 0.07 [b]
leucine	0.37 ± 0.02 [a]	0.41 ± 0.02 [a]	0.39 ± 0.03 [a]
lysine	1.45 ± 0.06 [b]	1.87 ± 0.06 [a]	1.23 ± 0.13 [c]
phenylalanine	1.39 ± 0.12 [a]	1.58 ± 0.07 [a]	1.44 ± 0.05 [a]
proline	1.99 ± 0.04 [b]	2.37 ± 0.10 [a]	1.70 ± 0.12 [c]
serine	2.53 ± 0.05 [a]	2.58 ± 0.08 [a]	2.60 ± 0.10 [a]
threonine	0.59 ± 0.02 [b]	0.66 ± 0.03 [a]	0.66 ± 0.04 [ab]
tyrosine	0.67 ± 0.00 [c]	1.03 ± 0.04 [a]	0.86 ± 0.05 [b]
valine	2.16 ± 0.03 [b]	2.51 ± 0.07 [a]	2.43 ± 0.05 [a]
**TOAs**, including:	10.00 ± 0.12 [b]	10.94 ± 0.41 [a]	8.45 ± 0.26 [c]
citric acid	2.34 ± 0.02 [a]	2.37 ± 0.08 [a]	1.66 ± 0.19 [b]
fumaric acid	0.22 ± 0.00 [a]	0.22 ± 0.01 [a]	0.16 ± 0.02 [b]
lactic acid	0.30 ± 0.04 [a]	0.18 ± 0.01 [b]	0.27 ± 0.07 [ab]
malic acid	6.15 ± 0.06 [b]	7.11 ± 0.25 [a]	5.31 ± 0.10 [c]
malonic acid	0.06 ± 0.00 [b]	0.07 ± 0.00 [a]	0.03 ± 0.01 [c]
oxalic acid	0.15 ± 0.00 [a]	0.16 ± 0.02 [a]	0.18 ± 0.04 [a]
propionic acid	0.11 ± 0.00 [a]	0.11 ± 0.01 [a]	0.12 ± 0.00 [a]
succinic acid	0.68 ± 0.02 [a]	0.70 ± 0.05 [a]	0.72 ± 0.07 [a]
**TRCs**, including:	9.57 ± 0.13 [b]	10.46 ± 0.37 [a]	9.70 ± 0.31 [b]
phosphoric acid	9.54 ± 0.14 [b]	10.42 ± 0.37 [a]	9.68 ± 0.30 [b]
urea	0.03 ± 0.00 [a]	0.04 ± 0.00 [a]	0.03 ± 0.01 [a]

Table S5. The concentration of total identified polar metabolites (**TIPMs**), including total soluble carbohydrates (**TSCs**), total amino acids (**TAAs**), total organic acids (**TOAs**), and total remaining compounds (**TRCs**) in **cotyledons** of 4-day-old seedlings of pea (*Pisum sativum* L.) cv. Nemo developed in water suspensions of bio-AgNPs at a concentration of 0, 50 and 200 mg/L. Values (in mg/g DW) are means of 3 replicates ± SD. The same letters by the values indicate no statistically significant ($p \le 0.05$) differences (valid separately for data in rows) based on ANOVA analysis and Tukey's post-hoc test.

		**bio-AgNPs**	
	**0 mg/L**	**50 mg/L**	**200 mg/L**
**Metabolites**		mg/g DW	
**TIPMs, including:**	**77.47 ± 2.14 [b]**	**87.76 ± 3.01 [a]**	**83.28 ± 1.33 [a]**
**TSCs**, including:	62.39 ± 1.96 [b]	71.93 ± 2.50 [a]	67.74 ± 1.12 [a]
fructose	0.09 ± 0.01 [a]	0.09 ± 0.01 [a]	0.10 ± 0.01 [a]
galactinol	0.06 ± 0.00 [b]	0.04 ± 0.00 [c]	0.08 ± 0.01 [a]
galactose	0.03 ± 0.00 [a]	0.03 ± 0.00 [b]	0.03 ± 0.00 [b]
glucose	0.05 ± 0.01 [b]	0.06 ± 0.01 [b]	0.11 ± 0.01 [a]
myo-inositol	1.97 ± 0.02 [b]	2.17 ± 0.08 [a]	1.89 ± 0.06 [b]
raffinose	0.07 ± 0.01 [b]	0.04 ± 0.01 [b]	0.12 ± 0.02 [a]
stachyose	0.14 ± 0.01 [a]	0.00 ± 0.00 [b]	0.21 ± 0.05 [a]
sucrose	59.80 ± 1.59 [b]	69.19 ± 2.45 [a]	65.05 ± 1.00 [a]
gluconic acid	0.17 ± 0.04 [b]	0.32 ± 0.06 [a]	0.15 ± 0.00 [b]
**TAAs**, including:	8.80 ± 0.28 [b]	9.31 ± 0.36 [ab]	9.53 ± 0.15 [a]
alanine	0.55 ± 0.02 [a]	0.44 ± 0.01 [b]	0.44 ± 0.02 [b]
asparagine	0.06 ± 0.00 [b]	0.07 ± 0.01 [b]	0.11 ± 0.01 [a]
aspartic acid	0.01 ± 0.00 [b]	0.01 ± 0.00 [b]	0.03 ± 0.00 [a]
β-alanine	0.09 ± 0.00 [ab]	0.09 ± 0.00 [b]	0.10 ± 0.00 [a]
GABA	2.28 ± 0.01 [a]	2.26 ± 0.10 [a]	1.99 ± 0.01 [b]
glutamic acid	0.72 ± 0.14 [b]	0.66 ± 0.15 [b]	0.96 ± 0.02 [a]
homoserine	1.49 ± 0.02 [b]	1.90 ± 0.09 [a]	1.53 ± 0.03 [b]
hydroxyproline	0.25 ± 0.03 [b]	0.28 ± 0.03 [ab]	0.31 ± 0.02 [a]
isoleucine	0.19 ± 0.01 [b]	0.22 ± 0.01 [a]	0.23 ± 0.01 [a]
leucine	0.11 ± 0.01 [b]	0.12 ± 0.02 [b]	0.15 ± 0.01 [a]
lysine	0.43 ± 0.02 [c]	0.54 ± 0.02 [b]	0.68 ± 0.02 [a]
phenylalanine	0.32 ± 0.02 [b]	0.36 ± 0.02 [a]	0.37 ± 0.01 [a]
proline	0.60 ± 0.02 [a]	0.56 ± 0.02 [a]	0.60 ± 0.02 [a]
serine	0.76 ± 0.02 [c]	0.81 ± 0.03 [b]	0.92 ± 0.01 [a]
threonine	0.05 ± 0.00 [a]	0.05 ± 0.00 [a]	0.05 ± 0.01 [a]
tyrosine	0.45 ± 0.01 [b]	0.46 ± 0.01 [b]	0.55 ± 0.02 [a]
valine	0.45 ± 0.01 [b]	0.49 ± 0.01 [a]	0.52 ± 0.01 [a]
**TOAs**, including:	4.28 ± 0.11 [a]	4.21 ± 0.11 [a]	3.92 ± 0.04 [b]
citric acid	1.90 ± 0.07 [b]	1.92 ± 0.10 [b]	2.36 ± 0.04 [a]
fumaric acid	0.04 ± 0.00 [b]	0.04 ± 0.00 [a]	0.04 ± 0.00 [b]
lactic acid	1.05 ± 0.03 [a]	0.84 ± 0.02 [b]	0.29 ± 0.01 [c]
malic acid	0.37 ± 0.01 [c]	0.45 ± 0.03 [b]	0.51 ± 0.01 [a]
malonic acid	0.13 ± 0.01 [b]	0.14 ± 0.00 [a]	0.15 ± 0.00 [a]
oxalic acid	0.17 ± 0.01 [a]	0.17 ± 0.03 [a]	0.16 ± 0.00 [a]
propionic acid	0.03 ± 0.00 [c]	0.04 ± 0.00 [b]	0.05 ± 0.00 [a]
succinic acid	0.59 ± 0.02 [a]	0.60 ± 0.04 [a]	0.36 ± 0.00 [b]
**TRCs**, including:	2.00[b] ± 0.07	2.33 ± 0.07 [a]	2.10 ± 0.03 [b]
phosphoric acid	1.95[b] ± 0.07	2.27 ± 0.07 [a]	2.09 ± 0.03 [b]
urea	0.05[a] ± 0.00	0.05 ± 0.01 [a]	0.01 ± 0.00 [b]
